# TAAR1-mediated pathways regulating nigrostriatal function and the discovery and pharmacological characterization of a novel TAAR1 agonist, Selutaront

**DOI:** 10.3389/fphar.2026.1759454

**Published:** 2026-02-23

**Authors:** Jianzhao Zhang, Xuan Deng, Jiawei Lv, Jing Lu, Lin Wang, Wenyan Wang, Hui Lei, Yunjie Wang, Jingwei Tian

**Affiliations:** 1 Key Laboratory of Molecular Pharmacology and Drug Evaluation (Yantai University), School of Pharmacy, Ministry of Education, Collaborative Innovation Center of Advanced Drug Delivery System and Biotech Drugs in Universities of Shandong, Yantai University, Yantai, China; 2 State Key Laboratory of Advanced Drug Delivery and Release Systems, Shandong Luye Pharmaceutical Co., Ltd., Yantai, China

**Keywords:** agonist, anti-schizophrenia, biological evaluation, regulatory network, trace amine-associated receptor 1

## Abstract

**Introduction:**

Schizophrenia is a severe neurodevelopmental disorder with limited treatment options. Trace amine-associated receptor 1 (TAAR1) has emerged as a promising therapeutic target, yet its downstream signaling mechanisms and the development of highly selective agonists remain incompletely explored.

**Methods:**

In this study, we established a TAAR1 knockdown rat model to elucidate its role in movement regulation and downstream signaling. Through molecular docking, site-directed mutagenesis and functional tests, we identified and characterized a novel TAAR1 agonist - Selutaront. We evaluated its antipsychotic-like effects in a mouse model similar to schizophrenia induced by MK-801 and identified its potential mechanism. Finally, we conducted pharmacokinetic analysis, Caco-2 permeability tests and liver microsomal stability tests to assess its drugability.

**Results:**

The research results indicate that TAAR1 mainly regulates the functional integrity of the substantia nigra-striatum circuit through the cAMP/PKA/CREB and DARPP32 signaling axes. Selutaront is a highly selective TAAR1 agonist, which forms a crucial interaction with the residues Asp103, Phe267, and Ser107 in the binding pocket. In vivo, Selutaront dose-dependently alleviates the schizophrenia-like behaviors induced by MK-801 in mice and counteracts the downregulation of the PKA/CREB pathway. Moreover, it exhibits excellent pharmacokinetic properties.

**Conclusion:**

This study demonstrates that TAAR1 regulates nigrostriatal function primarily via the PKA/CREB/DARPP32 signaling axis. Selutaront, a novel highly selective TAAR1 agonist with favorable drug-like properties, represents a promising candidate for schizophrenia treatment and provides mechanistic insights into TAAR1-targeted therapy.

## 1 Introduction

Schizophrenia is a severe neurodevelopmental psychiatric disorder of unknown aetiology, clinically characterised by positive symptoms (such as hallucinations and delusions), negative symptoms (such as emotional blunting and social withdrawal), and cognitive impairment (Marder and Cannon, 2019). According to the Global Burden of Disease Study (GBD 2019), the disorder ranks among the top ten causes of long-term disability globally. Its prevalence and incidence have risen steadily over decades, presenting an increasingly severe public health challenge (Solmi et al., 2023). Currently, the treatment of schizophrenia primarily involves pharmacological interventions (van Os and Kapur, 2009). While current first-line antipsychotic medications demonstrate efficacy against positive symptoms (Ye et al., 2025), their impact on negative symptoms and cognitive impairment remains limited (Huhn et al., 2019). Furthermore, these treatments are frequently associated with significant adverse effects, including metabolic abnormalities and weight gain (Patel et al., 2014; Pillinger et al., 2019; Kaar et al., 2020), leading to poor treatment adherence, high relapse rates (Robinson et al., 1999), and a considerable proportion of treatment-resistant cases (Lally et al., 2016; Demjaha et al., 2017). Consequently, developing novel therapeutic strategies with more comprehensive efficacy and enhanced safety profiles has become an urgent research priority.

In recent years, the trace amine-associated receptor 1 (TAAR1) has emerged as a prominent therapeutic target for psychiatric disorders due to its regulatory role within the central monoaminergic system (Dodd et al., 2021; Liu et al., 2024). TAAR1 is a predominantly intracellular G protein-coupled receptor (Borowsky et al., 2001) that can be activated by endogenous amines (such as β-phenylethylamine) (Gainetdinov et al., 2018) and exogenous substances (e.g., methamphetamine) (Borowsky et al., 2001; Liu et al., 2023). It exhibits extensive distribution across monoaminergic nuclei and associated projection areas in the mammalian brain (Rutigliano et al., 2017), with particular enrichment within dopaminergic, serotonergic, and glutamatergic neuronal populations. It plays a crucial role in reward, emotion, and cognitive functions (Lindemann et al., 2008; Berry et al., 2017; Alnefeesi et al., 2021), and through peripheral expression, participates in regulating metabolic processes such as improving glucose homeostasis and insulin secretion (Berry et al., 2017). At the signalling mechanism level, TAAR1 can couple to the Gαs protein-cAMP-PKA pathway (Borowsky et al., 2001; Bunzow et al., 2001; Miller et al., 2005; Xie et al., 2007; Bradaia et al., 2009; Panas et al., 2011) and also influence the AKT/GSK-3β cascade via β-arrestin 2 (Harmeier et al., 2015). However, the precise mechanisms of TAAR1 in specific brain regions and behavioural phenotypes remain incompletely elucidated, and its complex signalling networks warrant further investigation.

Given their modulatory properties and metabolic benefits, TAAR1 agonists are regarded as a novel strategy to circumvent the adverse effects of conventional antipsychotic drugs, such as metabolic disorders and extrapyramidal reactions (Shang et al., 2023; Siafis et al., 2024; Smith et al., 2025). Beyond Ulotaront (SEP-363856), a TAAR1/5-HT_1A_ dual agonist (Koblan et al., 2020), other candidates such as Ralmitaront (Ågren et al., 2023), a partial TAAR1 agonist, have demonstrated potential in preclinical and early clinical studies. Ulotaront, in particular, demonstrated robust efficacy in Phase II studies with minimal metabolic or extrapyramidal side effects (Achtyes et al., 2023), although subsequent Phase III trials (DIAMOND 1 and 2) did not meet primary endpoints due to high placebo responses (Siafis et al., 2024; Smith et al., 2025). These dual-targeted drugs are unable to clearly delineate the independent contribution of TAAR1 activation, and highly selective TAAR1 agonists remain unavailable on the market. Therefore, the development and characterization of highly selective TAAR1 agonists are essential to elucidate the precise mechanisms underlying TAAR1-mediated antipsychotic effects and to fully realize the therapeutic potential of this novel target.

Based on this background, the present study was designed to systematically accomplish the entire process, from target validation to the discovery and comprehensive characterization of a novel TAAR1 agonist. Multiple dopaminergic pathways have been identified as being involved in the symptoms and pathology of schizophrenia; all these pathways originate in the midbrain (ventral tegmental area or substantia nigra) (Correll et al., 2022). Among these, increased dopamine in the associative striatum is considered to be the basis for positive symptoms (Howes et al., 2009; Kegeles et al., 2010). In the ventral striatum, low levels of dopamine are associated with negative symptoms (Radua et al., 2015). First, we established a rat model with targeted knockout of TAAR1 in the substantia nigra and striatum regions to investigate the direct effects of TAAR1 deficiency on animal behavior and its downstream molecular events, which may provide genetic evidence for the core physiological functions of TAAR1. Then, building upon these findings, a novel TAAR1 agonist, Selutaront, was designed and synthesized. We systematically evaluated its TAAR1 agonist activity, receptor selectivity, and efficacy in reducing hyperactivity in MK801-induced schizophrenia-like mice. Moreover, the molecular interaction mechanism between Selutaront and human TAAR1 was thoroughly elucidated. Finally, the pharmacokinetic profile and *in vitro* metabolic stability of Selutaront were systematically assessed to evaluate its potential as a preclinical candidate.

## 2 Results

### 2.1 Region-specific knockdown of TAAR1 in the substantia nigra and striatum of rats

**FIGURE 1:**
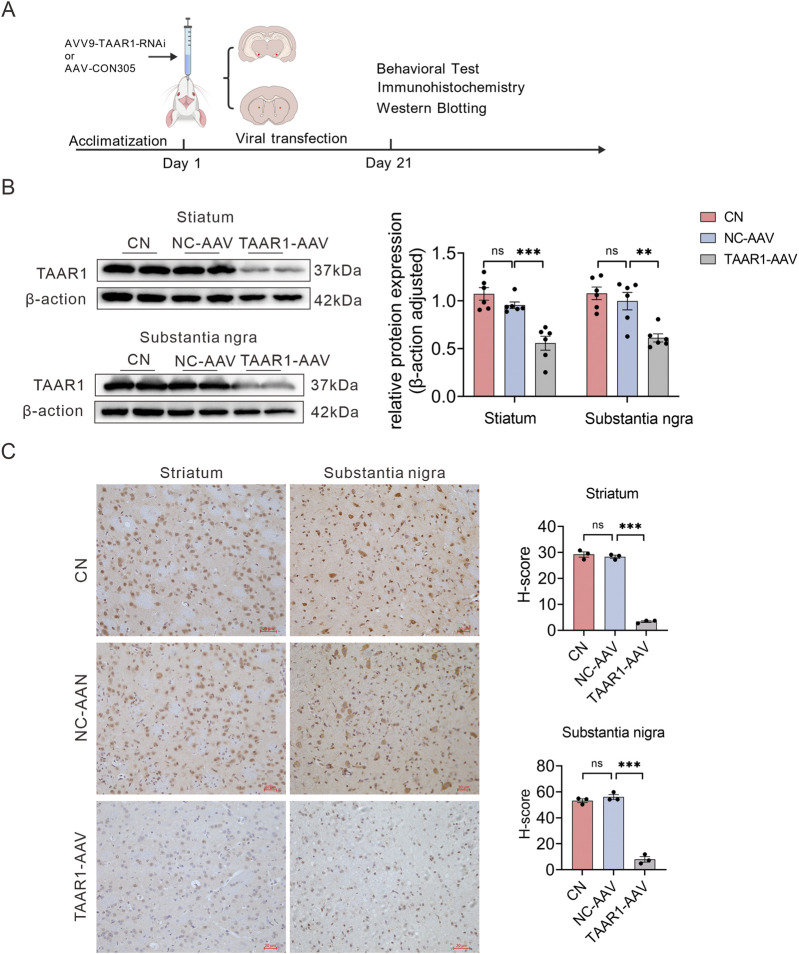
Validation of the efficiency of TAAR1 gene knockdown. **(A)** Experimental schematic: Bilateral stereotaxic injection of TAAR1-AAV into rat striatum and substantia nigra, followed by behavioral testing and tissue collection after a 3-week transfection period (Created with BioGDP.com (Jiang et al., 2025)). **(B)** Western blot quantification: TAAR1 protein expression was significantly downregulated in striatal and substantia nigra tissues of TAAR1-AAV treated rats compared to NC-AAV groups (Data are presented as mean ± SEM, n = 6, **P < 0.01, ***P < 0.001). **(C)** Representative immunohistochemical images: Markedly reduced TAAR1 immunoreactivity in striatal and substantia nigra regions of the TAAR1-AAV group versus the NC groups. Scale bar: 20 μm (Data are presented as mean ± SEM, n = 3, ***P < 0.001).

To investigate the role of TAAR1 in motor regulation and related neural circuits, an adeno-associated virus (AAV) carrying TAAR1-specific shRNA was delivered bilaterally into the striatum and substantia nigra of Sprague-Dawley (SD) rats via stereotaxic injection to establish a TAAR1 knockdown (TAAR1-KD) rat model (Figure 1A). Western blotting and immunohistochemistry (IHC) analyses revealed that TAAR1 protein expression in both the striatum and substantia nigra was significantly reduced in the TAAR1-AAV group compared to the Control (CN) and negative control (NC-AAV) groups (*P* < 0.001; Figures 1B,C), indicating successful model establishment.

### 2.2 TAAR1 knockdown in striatum and substantia nigra induced hyperactivity

**FIGURE 2:**
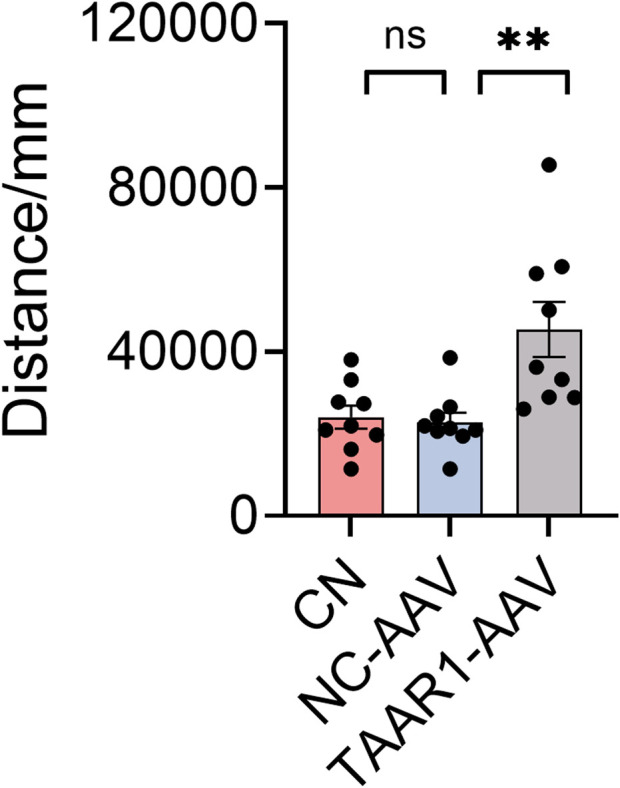
Results from the open field test showed that the TAAR1-AAV group exhibited increased locomotor activity compared to NC-AAV group (Data are presented as mean ± SEM, n = 9, **P < 0.01).

To assess whether the knockdown of TAAR1 in substantia nigra and striatum induces schizophrenia-like behavior, spontaneous locomotor activity was measured in TAAR1-KD rats using the open field test (OFT). The results showed that spontaneous locomotor activity was significantly increased in the TAAR1-KD group compared to the NC-AAV group (Wolinsky et al., 2006) (*P* < 0.01; Figure 2), suggesting that TAAR1 may be involved in modulating motor behavior within the nigrostriatal circuit. This finding provides a behavioral basis for further investigation into the role of TAAR1 in schizophrenia-related motor phenotypes and underlying neural mechanisms.

### 2.3 TAAR1 in the striatum and substantia nigra regulates the PKA/DARPP32 and PKA/CREB pathway

TAAR1 is a G protein-coupled receptor known to couple to Gs and Gq proteins (Xu et al., 2023), whose activation is implicated in modulating protein kinase A (PKA) and protein kinase B (AKT)/Glycogen Synthase Kinase-3β (GSK-3β)pathway (Xie and Miller, 2007; Espinoza et al., 2015).

**FIGURE 3:**
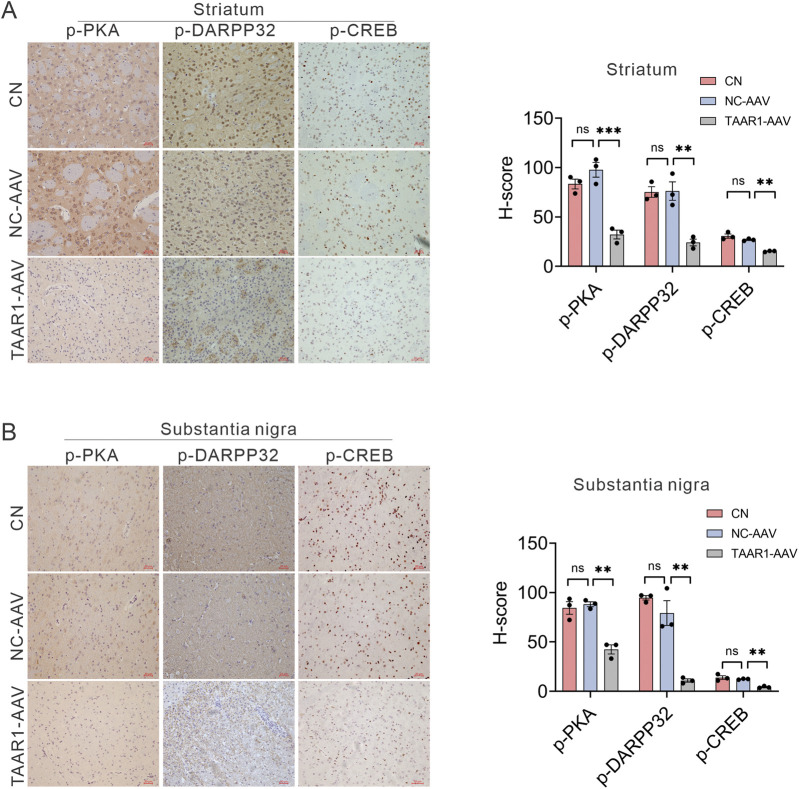
Knockdown of TAAR1 affects the expression of p-PKA, p-DARPP32, and p-CREB. Representative immunohistochemical staining and quantitative analysis of the striatum **(A)** and substantia nigra. **(B)** From TAAR1-AAV treated rats, showing significantly reduced phosphorylation levels of DARPP-32 (Thr34), CREB (Ser133), and PKA (Thr197) (Data are presented as mean ± SEM, n = 3, **P < 0.01, ***P < 0.001).

To further investigate the *in vivo* signaling mechanisms of TAAR1, key signaling molecules were systematically examined in the TAAR1-KD rat model. Immunohistochemistry (IHC) analysis revealed that the phosphorylation levels of protein kinase A (PKA) (Zhang et al., 2018), cAMP Response Element-Binding Protein (CREB) (Wang et al., 2018), and Dopamine- and cAMP-regulated neuronal phosphoprotein 32 kDa (DARPP32) (which can be phosphorylated by PKA at Thr34) (Matsuyama et al., 2002) were significantly reduced in the striatal tissue of TAAR1-KD rats compared to NC-AAV group (Figure 3A), indicating that TAAR1 deficiency impairs its canonical cAMP/PKA signaling pathway and downstream regulatory network. In the substantia nigra, a similar reduction in the phosphorylation levels of PKA, CREB, and DARPP-32 was observed (Figure 3B), which was consistent with the changes observed in the striatum.

**FIGURE 4:**
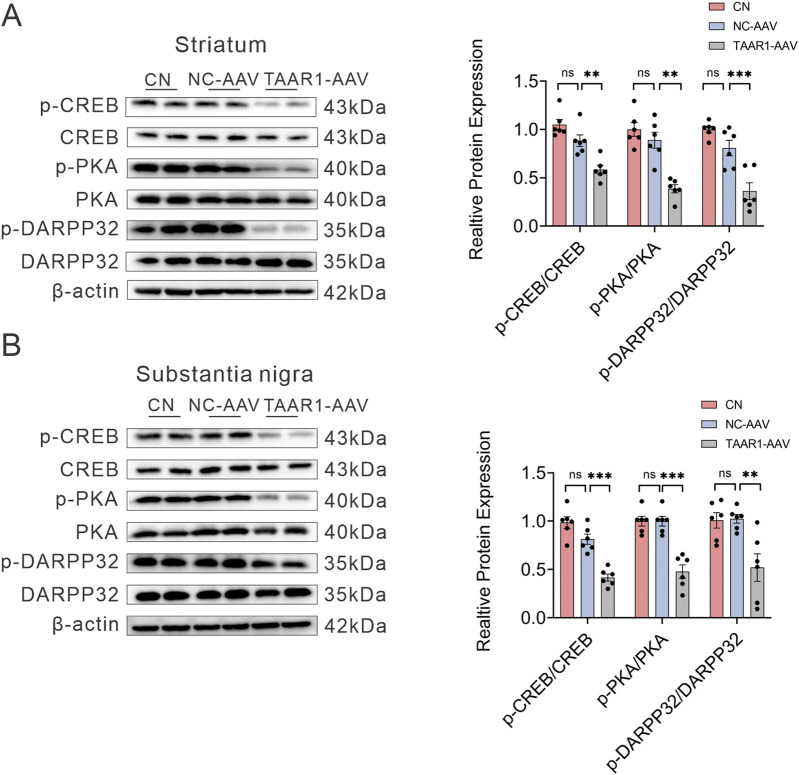
Representative bands of Western blot and quantitative analysis. Deficiency of TAAR1 in striatum **(A)** and substantia nigra **(B)** affects the protein expression of p-PKA, p-DARPP32, and p-CREB (Data are presented as mean ± SEM, n = 6, **P < 0.01, ***P < 0.001).

Since IHC results revealed a significant downregulation of the PKA pathway in the striatum and substantia nigra following TAAR1 knockdown, brain tissues from the striatum and substantia nigra were selected for Western blot validation. The results demonstrated that, compared NC-AAV group, the phosphorylation levels of PKA, CREB, and DARPP-32 were all significantly reduced in the striatal tissues of TAAR1-KD rats (Figure 4).

Notably, the total and phosphorylated protein levels of AKT and GSK-3β showed no significant differences between TAAR1-AAV and NC-AAV groups (see Supplementary Figure S1), suggesting that TAAR1 knockdown did not significantly alter the activity of the AKT/GSK-3β pathway in the striatum. Furthermore, the expression of other relevant signaling molecules, including ERK1/2 (Shi et al., 2019), MEK1/2 (Michael et al., 2019), and BDNF (Zheng et al., 2023), was examined, and no significant alterations were observed.

### 2.4 Selutaront acts as a selective TAAR1 agonist, exhibiting no detectable agonist activity at dopaminergic or serotonergic (5-HT) receptors

We designed and synthesised Selutaront, characterising and validating it via proton nuclear magnetic resonance (^1^HNMR), high-performance liquid chromatography (HPLC), and liquid chromatography-mass spectrometry (LC-MS) (see Supplementary Figures S2–S4).

**FIGURE 5:**
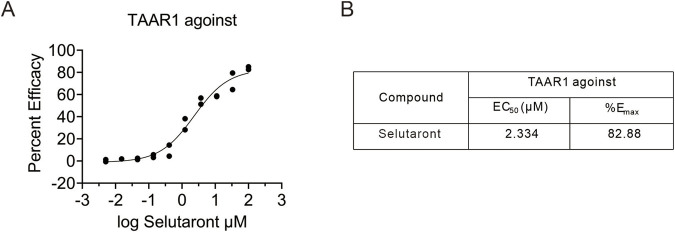
Functional activation of human TAAR1 by Selutaront. **(A,B)** cAMP levels were measured in CHO-K1 AGTRL1 Gi cells stably expressing human TAAR1 to evaluate the TAAR1 agonist activity of Selutaront. Data represent three independent experiments.

The Selutaront exhibited potent TAAR1 agonist activity in stably transfected human TAAR1-expressing cells, with an EC_50_ of 2.33 μM and an E_max_ of 83% (Figure 5). This confirms that Selutaront is a potent TAAR1 agonist. To assess target specificity, agonist/antagonist activity of Selutaront was evaluated against schizophrenia-relevant receptors (namely 5-HT_1A_, D_2L_, and D_2S_). Functional assays revealed no significant agonist or antagonist activity at concentrations up to 100 μM (EC_50_/IC_50_ > 100 μM) for these receptors (Table 1). These findings demonstrate that Selutaront is a highly selective TAAR1 agonist.

**TABLE 1:** Agonist and antagonist activities of Selutaront at 5-HT_1A_, dopamine D_2L_, and D_2S_ receptors.

Compound	5HT_1A_ assay		D_2L_				D_2S_	
	Agonist		Antagonist		Agonist		Antagonist	
	EC_50_ (μM)	%E_max_	IC_50_(μM)	%Inh	EC_50_ (μM)	%E_max_	IC_50_(μM)	%Inh
Selutaront	>100	4.52	>100	−8.70	>100	16.23	>100	−34.87

### 2.5 Molecular interactions of Selutaront with TAAR1 and functional residue validation

**FIGURE 6:**
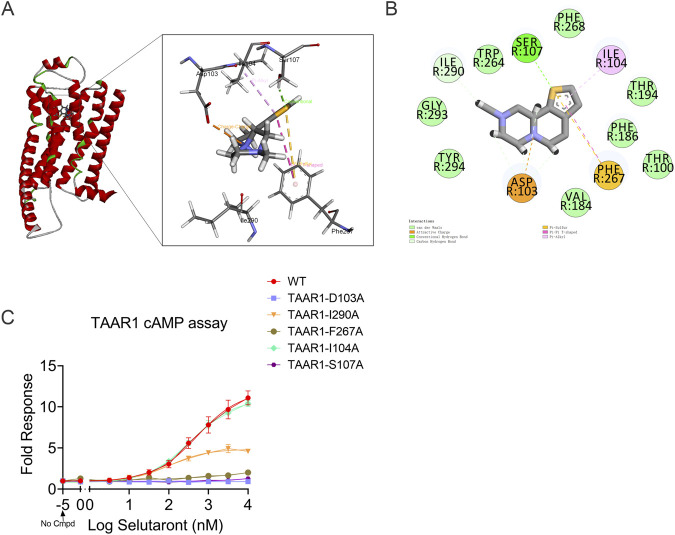
Binding Mechanism Analysis of Selutaront to TAAR1. **(A,B)** Molecular docking of the TAAR1- Selutaront complex: Left: 3D representation of the binding pocket; Right: 2D interaction diagram between Selutaront and key TAAR1 residues. **(C)** Functional validation of TAAR1 mutants: cAMP accumulation induced by Selutaront in TAAR1 mutants was measured using the GloSensor assay system. Data represent mean ± SEM of three independent experiments.

Molecular docking simulations were conducted using the human TAAR1 crystal structure (PDB ID: 8JLO) in Discovery Studio to elucidate the structural mechanism underlying Selutaront-TAAR1 interactions. The simulations revealed stable binding of Selutaront within the TAAR1 orthosteric pocket through multiple interactions (Figures 6A,B): The protonated amino group formed an attractive charge with Asp103, which determines primary binding orientation. Its thiophene moiety engaged in dual π-sulfur and T-shaped π-π interactions with Phe267. A conventional hydrogen bond occurred with Ser107 via the thiophene sulfur atom. A carbon-hydrogen bond formed between the methyl group and Ile290, with additional π-alkyl interactions between the thiophene ring and Ile104. To validate computational predictions, TAAR1 point mutants were generated for functional analysis (Figure 6C). Mutations of critical residues (D103A/F267A/S107A) nearly abolished Selutaront-mediated TAAR1 activation, confirming their essential role in ligand recognition. Mutations of fine-tuning residues (I104A, I290A) reduced cAMP accumulation, indicating their involvement in binding energy optimization and conformational modulation. These findings provide precise structure-based targeting strategies for developing high-selectivity TAAR1 agonists.

### 2.6 Selutaront ameliorates MK-801-induced schizophrenia-like behaviors via the TAAR1 pathway

**FIGURE 7:**
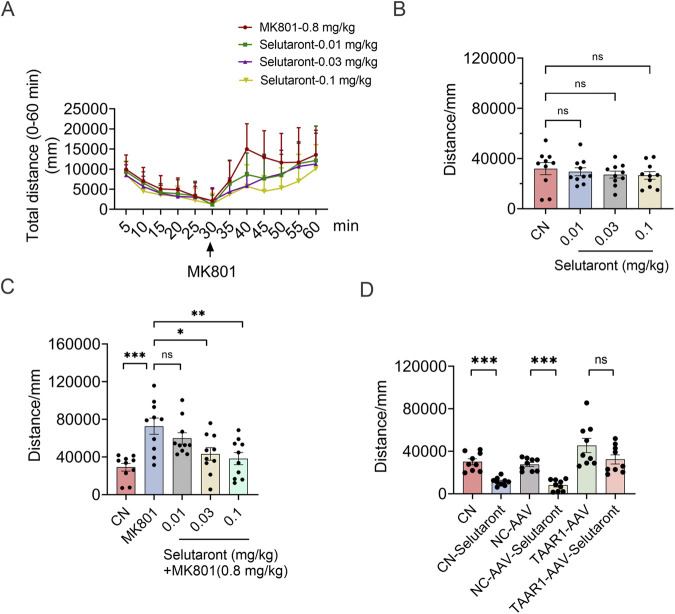
Selutaront Ameliorates MK-801-Induced Schizophrenia-Like Behaviors via the TAAR1 Pathway. **(A–C)** Oral administration of Selutaront dose-dependently attenuated MK-801-induced schizophrenia-like behaviors in C57BL/6J mice (Data are presented as mean ± SEM, n = 10, *P < 0.05, **P < 0.01, ***P < 0.001, ns: not significant: *P* > 0.05). **(D)** Open field test (OFT) results: Selutaront failed to reduce spontaneous locomotor distance in the TAAR1-knockdown group (*P* > 0.05), whereas significant motor suppression was observed in Selutaront-treated Sham and NC groups (Data are presented as mean ± SEM, n = 9, ***P < 0.001).

To evaluate the antipsychotic effects of Selutaront, behavioral tests were conducted in a C57BL/6J mouse model. Thirty minutes after a single oral administration of Selutaront (0.01, 0.03, or 0.1 mg/kg), schizophrenia-like behaviors were induced via an intraperitoneal injection of the NMDA receptor antagonist MK-801 (0.8 mg/kg) (Kruk-Slomka et al., 2017; Fan et al., 2018). The results demonstrated that Selutaront (0.03, 0.1 mg/kg) did not affect the animals’ spontaneous activity and exhibited a dose-dependent suppression of MK-801-induced schizophreniform behavioural abnormalities (Figures 7A–C), confirming its efficacy in alleviating core positive symptoms of schizophrenia. To investigate the mechanism of action, a TAAR1 partial knockdown model was subsequently established by stereotaxically injecting a TAAR1- AAV virus into the striatum and substantia nigra of SD rats. The open field test showed that TAAR1 knockdown resulted in increased locomotor activity in rats (Figure 2). However, following oral administration of Selutaront (5 mg/kg), the expected suppression of activity observed in the control group was absent in the knockdown group (Figure 7D). This finding indicates that the antipsychotic-like behavioral effects of Selutaront are dependent on an intact TAAR1 signaling pathway, providing key mechanistic evidence for its potential as a targeted therapy for schizophrenia.

### 2.7 Selutaront exerts regulatory effects on the PKA/CREB pathway

**FIGURE 8:**
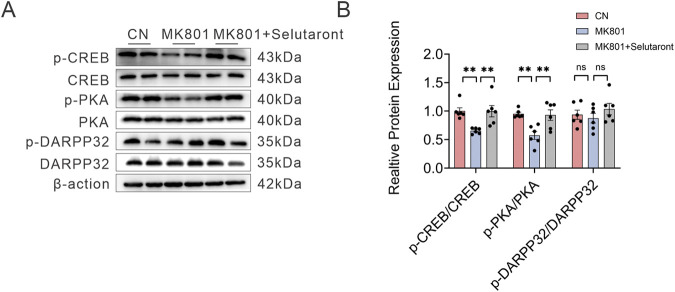
**(A)** Representative Western blot bands showing protein expression levels of p- DARPP-32 (Thr34), total DARPP-32, p-PKA (Thr197), total PKA, p-CREB (Ser133), total CREB, and the internal reference β-actin. **(B)** Quantitative analysis of the relative expression ratios of p-DARPP-32/DARPP32, p-PKA/PKA and p-CREB/CREB (both normalized to β-actin). Data are presented as mean ± SEM, n = 6, **P < 0.01, ns: not significant, *P* > 0.05.

The above findings suggested that TAAR1 exerted regulatory effects on the PKA/DARPP32 and PKA/CREB pathways. To further investigate whether this regulation is mediated by TAAR1 agonism, we treated MK-801-induced hyperactive mice with the TAAR1 agonist Selutaront. As shown in Figure 8, compared to the Control group, administration of MK-801 (0.8 mg/kg) alone significantly reduced the phosphorylation levels of p-PKA (Thr197) and p-CREB (Ser133) (*P* < 0.01). In contrast, pretreatment with Selutaront (10 mg/kg) significantly reversed the MK-801-induced decrease in p-PKA and p-CREB levels (*P* < 0.01 vs. the MK-801 group), restoring their phosphorylation to levels that showed no statistically significant difference from the Control group (*P* > 0.05). These results confirm that by functionally antagonizing the effects of MK-801, Selutaront molecularly rescues and restores the suppressed TAAR1 downstream cAMP-PKA-CREB signaling pathway, thereby providing direct mechanistic evidence for its ameliorative effects on schizophrenia-like behaviors.

### 2.8 Pharmacokinetic profile of Selutaront

**FIGURE 9:**
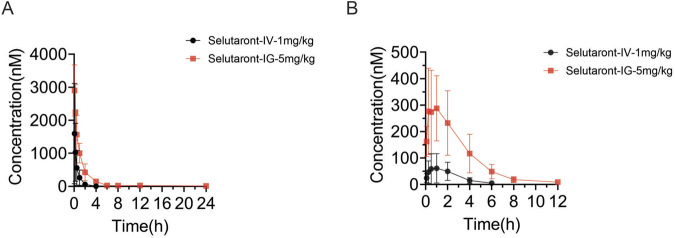
Pharmacokinetics of Selutaront in Male Sprague-Dawley Rats (n = 3). **(A)** Mean plasma concentration-time curves of the parent drug (Selutaront) following a single intravenous (IV) injection (1 mg/kg) or oral gavage (5 mg/kg) administration in male rats. **(B)** Mean plasma concentration-time curves of metabolites following a single IV injection (1 mg/kg) or oral gavage (5 mg/kg) of Selutaront in male rats.

The above findings thoroughly analyze the downstream pathways mediated by TAAR1 and the therapeutic effects of its agonist (Selutaront) on schizophrenia. To evaluate the drug potential of Selutaront, we analyzed its pharmacokinetic behavior *in vivo* and *in vitro* (Table 2). Pharmacokinetic studies in rats revealed favorable oral absorption and systemic exposure characteristics for Selutaront. Following oral administration (5 mg/kg), the following parameters were observed: time to maximum concentration (T_max_) = 0.139 h, the terminal elimination half-life (T_1/2_) = 3.46 h, area under the concentration time curve (AUC) = 4,582 h·ng/mL, and absolute oral bioavailability (F) = 48.1% (Figure 9).

**TABLE 2:** Pharmacokinetic profile of Selutaront in male rats.

Subject	T_max_ (h)	C_max_ (nM)	AUC_last_ (h·nM)	T_1/2_ (h)	Vss_obs (L/kg)	Cl_obs (L/h/kg)	MRT_last_ (h)	F (%)
Selutaront - IV 1 mg/kg	0.083 ± 0	3,113 ± 81	1906 ± 46	0.528 ± 0.054	1.46 ± 0.06	2.51 ± 0.06	0.566 ± 0.005	—
Selutaront - IG 5 mg/kg	0.139 ± 0.096	3,747 ± 2011	4,582 ± 1,321	3.46 ± 4.30	—	—	1.89 ± 1.15	48.1 ± 10.3

Selutaront exhibited favorable membrane permeability and metabolic stability (Tables 3, 4). It demonstrated high permeability (P_app_ A→B = 36.0 × 10^−6^ cm/s) and low efflux risk (efflux ratio [ER] = 0.788, significantly below the threshold of 2.0), indicating minimal risk of efflux transporter-mediated intestinal efflux. Selutaront displayed substantially greater metabolic stability in human liver microsomes compared to rodent liver microsomes: 103% remaining at 60 min, half-life (T_1/2_) >186 min, and intrinsic clearance (CL_int_) <6.75 mL/min/kg (low clearance). The human hepatic intrinsic clearance was less than one-sixth of the murine value (human: <6.75 vs. mouse: 43.6 mL/min/kg), with the half-life exceeding 1.5-fold that in mice (human: >186 vs. mouse: 126 min). These properties collectively indicate favorable oral absorption potential in humans, likely resulting in low hepatic first-pass effect, extended *in vivo* half-life, and high oral bioavailability, providing crucial pharmacokinetic advantages for clinical development.

**TABLE 3:** Permeability and efflux ratio assessment of Selutaront in Caco-2 cell monolayers.

Caco-2 permeability	Mean P_app_ (10^−6^ cm/s)		Efflux ratio	Rank	
	A to B	B to A		Papp	Efflux transporter substrate
Selutaront	36.0	28.4	0.788	High	Poor or non-

**TABLE 4:** *In Vitro* metabolic stability parameters of Selutaront in hepatic microsomes across species.

Compound	Species	% remaining (%, T = 60 min)	T_1/2_ (min)	CL_int_ (mic) (μL/min/mg)	CL_int_ (liver) (mL/min/kg)	Clearance classification
Selutaront	Mouse	74.7	126	11.0	43.6	Medium
	Rat	68.2	99.0	14.0	25.2	Medium
	Human	103	>186	<7.50	<6.75	Low

## 3 Discussion

The current pharmacotherapy for schizophrenia primarily relies on antipsychotic medications that predominantly act through dopamine D_2_ receptor antagonism (FGAs) or dual serotonin-dopamine antagonism (SGAs) (Abi-Dargham and Laruelle, 2005; Carbon et al., 2018). However, they are associated with substantial side effects. FGAs frequently cause extrapyramidal symptoms and tardive dyskinesia, whereas SGAs often lead to metabolic syndrome, including weight gain, dyslipidemia, and increased diabetes risk (Carbon et al., 2017; Kaar et al., 2020). Approximately 30% of patients exhibit treatment resistance, with clozapine being the only effective option yet burdened by potentially severe adverse effects such as agranulocytosis and the need for stringent monitoring.

These limitations underscore the urgent need for novel therapeutic agents with different mechanisms of action. TAAR1 agonists represent a promising class of investigational antipsychotics that modulate presynaptic dopamine function rather than blocking postsynaptic D_2_ receptors. Preclinical and clinical studies, including ulotaront (SEP-363856), demonstrate that TAAR1 activation significantly reduces striatal dopamine synthesis capacity, correlating with improvements in positive symptoms without inducing extrapyramidal side effects or metabolic disturbances (Nair et al., 2021; Højlund and Correll, 2022). TAAR1 agonists offer a groundbreaking therapeutic approach that directly targets the dopaminergic pathophysiology of schizophrenia. Their distinct mechanism holds promise for effectively treating a broad range of symptoms, including the often-neglected negative and cognitive domains, while minimizing the debilitating side effects associated with current antipsychotics, thereby addressing critical unmet needs in schizophrenia management.

TAAR1 is widely distributed in the mammalian brain, with high density observed in limbic and monoaminergic regions, where it modulates processes such as emotion, attention, memory, fear, and addiction (Xie and Miller, 2009). Nevertheless, its precise functional roles remain poorly defined. Several studies have investigated alterations in trace amine (TA) levels in biofluids, including urine, cerebrospinal fluid, and plasma, in relation to schizophrenia (Boulton, 1982; Egerton et al., 2013; John et al., 2017). It is noteworthy that recent studies examining post-mortem brain tissue from individuals diagnosed with schizophrenia have yielded substantial evidence in support of the notion that TAAR1 plays a direct role in the etiopathogenesis of this disorder. For instance, research has revealed the presence of elevated levels of mRNA and protein forms of TAAR1 in the brain tissue of patients (Imbriglio et al., 2024). As indicated by previous studies, there is a demonstrable correlation between TAAR1 single nucleotide variants/polymorphisms and neuropsychiatric disorders, including schizophrenia (Shajan et al., 2024). Collectively, these findings establish TAAR1 as a credible molecular target within the disease mechanism. Furthermore, it has garnered significant attention for its ability to inhibit dopaminergic neuron overactivation, balance glutamate-GABA signaling, and enhance synaptic plasticity (Bradaia et al., 2009; Revel et al., 2011; Zhang et al., 2021; Zhang et al., 2024). However, its precise mechanisms of action within specific brain regions have not been fully elucidated.

This study involved the first construction of a TAAR1 partial knockdown rat model, providing a critical tool for understanding the role of TAAR1 in schizophrenia-related behaviors. Previous studies have indicated that TAAR1-KO mice exhibit a characteristic positive symptom of schizophrenia—sensorimotor gating deficits (Wolinsky et al., 2006). It was observed that TAAR1 knockdown induced a significant increase in spontaneous locomotor activity in rats, a behavioral phenotype closely associated with positive symptoms and hyperkinesia in schizophrenia (Jones et al., 2011; Winship et al., 2018). Furthermore, in the striatum and substantia nigra of knockdown rats, the phosphorylation levels of key signaling molecules, including p-PKA (Thr197), p-DARPP-32 (Thr34), and p-CREB (Ser133), were coordinately downregulated. This suggests that the behavioral abnormalities resulting from TAAR1 deficiency may be directly linked to the suppression of the cAMP/PKA signaling axis, thereby linking TAAR1 function to schizophrenia-related behavioral phenotypes at a molecular level. Notably, pathways such as AKT/GSK-3β were not significantly altered following TAAR1 knockdown, further supporting the conclusion that TAAR1 modulates neural function primarily via the cAMP/PKA signaling axis within the nigrostriatal circuit.

Based on an in-depth understanding of TAAR1 function, a novel TAAR1 agonist, Selutaront, was developed. In cells stably expressing human TAAR1, Selutaront demonstrated potent agonist activity (EC_50_ = 2.33 μM, Emax = 83%) and showed no significant activity at the dopamine D_2_ or 5-HT_1A_ receptors, confirming its high selectivity as a TAAR1 agonist. This profile thereby effectively avoids the adverse effects associated with D_2_ receptor engagement by traditional antipsychotics, such as extrapyramidal symptoms and hyperprolactinemia (Siafis et al., 2023), and circumvents the complex pharmacodynamic issues linked to 5-HT_1A_ receptor activation (Yamada et al., 2023). Furthermore, through molecular docking and site-directed mutagenesis experiments, the binding mechanism of Selutaront to TAAR1 was elucidated, identifying critical residues such as Asp103 and Phe267/Ser107 that play vital roles in ligand recognition and providing precise targets for structure-based drug optimization.

The highly selective activation of TAAR1 by Selutaront suggests its potential as an antipsychotic drug candidate. Accordingly, the antipsychotic efficacy of Selutaront was evaluated in a C57BL/6J mouse model. Specifically, Selutaront was observed to reverse MK-801-induced hyperlocomotion in wild-type animals at doses (0.1–0.3 mg/kg) that did not affect their normal spontaneous activity. However, its behavioral effects were largely abolished in TAAR1 partial knockdown rats. This result provides the most direct evidence for the target specificity of this compound. Further mechanistic studies revealed that Selutaront could reverse the MK-801-induced decrease in the phosphorylation levels of PKA and CREB. Therefore, it is hypothesized that the pharmacological effects of Selutaront are mediated via the TAAR1-cAMP-PKA-CREB signaling axis, thereby modulating dopaminergic circuitry.

Selutaront demonstrated favorable pharmacokinetic properties: oral bioavailability of 48.1% with rapid absorption onset (T_max_ = 0.139 h). Excellent metabolic stability was observed in human liver microsomes (T_1/2_ > 186 min, CL_int_ < 6.75 mL/min/kg), significantly greater than in rodents (Table 2), indicating a low first-pass effect and an extended half-life. High Caco-2 permeability (P_app_ (A→B) = 36.0 × 10^−6^ cm/s) without efflux transporter liability (ER = 0.788) was confirmed (Table 1). These characteristics address the common limitations of neuropsychiatric drugs, low oral bioavailability and rapid metabolism, thereby laying the foundation for clinical development.

Despite the significant advances achieved in this study, certain limitations remain. For instance, in the TAAR1 partial knockdown rats, the analysis of altered downstream signaling molecules was primarily concentrated in the striatum, whereas in the substantia nigra, only PKA, CREB, and DARPP-32 were examined. The MK-801 model primarily mimics the positive symptoms of schizophrenia; consequently, the ameliorative effects of Selutaront on negative symptoms and cognitive impairment require further evaluation in more comprehensive animal models. Additionally, the safety and toxicological profile following long-term administration warrants further investigation.

In summary, this study not only explored the downstream signaling pathways of TAAR1 in the striatonigral circuit, more importantly, led to the development of Selutaront, a TAAR1 agonist characterized by high selectivity, a well-defined mechanism of action, and favorable drug-like properties. These findings provide compelling preclinical evidence for the development of a new generation of antipsychotic drugs and offer novel promise for overcoming the current limitations in schizophrenia treatment.

## 4 Materials and methods

### 4.1 Drugs and chemicals

Selutaront was provided by WuXi AppTec (Shanghai, China). (+)-MK-801 hydrogen maleate was purchased from Sigma-Aldrich (St. Louis, MO, United States). Fetal Bovine Serum (FBS, Value-Added) and Trypsin-EDTA (0.25%) solution were obtained from Gibco™, Thermo Fisher Scientific (Waltham, MA, United States). The HitHunter® cAMP Assay for Small Molecules kit was sourced from Eurofins Discovery (Fremont, CA, United States), and the FLIPR® Calcium 6 Assay Kit was from Molecular Devices (San Jose, CA, United States). Cell culture media, including Ham’s F-12K (Kaighn’s) Medium and MEM (Minimum Essential Medium), were supplied by Invitrogen (Camarillo, CA, United States). Antibiotics (Penicillin, Streptomycin, and Hygromycin B) were purchased from Beyotime Biotechnology (Shanghai, China).

The TAAR1-targeting shRNA adeno-associated virus (AAV9-TAAR1-shRNA; target sequence: 5′-GCG​CCA​CAA​AGC​AAG​GAA​ACA-3′) and the control virus (AAV9-U6-scramble-shRNA-EGFP) were provided by GeneChem (Shanghai, China) at a titer of 1.50 × 10^13^ vector genomes (vg)/mL.

### 4.2 Animals

Male Sprague-Dawley rats (6–8 weeks; 190–210 g) and male C57BL/6J mice (6–8 weeks; 18–22 g) were procured for the purposes of this study by Vital River Company (Beijing, China). All animals resided under specific pathogen-free (SPF) conditions in a climate-controlled environment with a 12-h light/dark cycle (8:00–20:00), constant temperature of 22 °C ± 2 °C, and 40%–70% humidity. Standard rodent diet and water were provided *ad libitum*. All animals underwent a 1-week habituation period before experimentation. All behavioral assessments occurred between 08:00 and 14:00 h.

All animal experiments described in this manuscript were conducted in accordance with relevant guidelines and regulations. The study was jointly approved by the Laboratory Animal Care and Use Committee of Yantai University and the Laboratory Animal Care and Use Committee of Shandong Luye Pharmaceutical Group.

### 4.3 Stereotactic surgery for targeted TAAR1 knockdown

Adult male Sprague-Dawley rats were anesthetized via intraperitoneal (i.p.) injection of sodium pentobarbital (40 mg/kg; prepared in sterile 0.9% saline) and subsequently immobilized in a Stoelting stereotaxic apparatus (Model 51700D, Stoelting Co., Wood Dale, IL, United States). Bilateral injections targeting striatum and substantia nigra were performed using coordinates relative to bregma (Paxinos and Watson, 6th ed.) (Paxinos and Watson, 2006): Striatum: Antero-posterior (AP): +0.43 mm, Medio-Lateral (ML): ±2.7 mm, Dorso-Ventral (DV): −5.4 mm; Substantia Nigra: Antero-Posterior (AP): −5.0 mm, Medio-Lateral (ML): ±2.1 mm, Dorso-Ventral (DV): −8.5 mm. Viruses (2 μL/site) were infused at 0.3 μL/min via a Hamilton microsyringe. The needles were left in place for a period of 5 minutes following the infusion before being withdrawn. Surgical incisions were sutured, and penicillin G (50,000 IU/kg, diluted in saline, i.p.) was administered as infection prophylaxis. Animals were singly housed for 3 weeks to permit maximal viral expression, with daily monitoring for surgical recovery and health status.

### 4.4 Open field test (OFT)

Prior to testing, animals were habituated in the behavioral testing room for ≥2 h. The rats were individually positioned within an open-field arena (50 × 50 × 40 cm; length × width × height) connected to the TopScan® behavioral monitoring system (Clever Sys Inc., Reston, VA, United States). Testing was conducted under low illumination (≤50 lux) with minimal ambient noise (<50 dB). Total distance traveled (mm) over 30 min was recorded and analyzed using the TopScan system. After each experimental group, feces were removed, and the arena was wiped with 75% alcohol to eliminate potential confounding odors.

### 4.5 Western blotting

Striatal and substantia nigra tissues were isolated from Sprague-Dawley (SD) rats transfected with TAAR1-AAV (n = 6). Tissues were flash-frozen in liquid nitrogen and homogenized in RIPA lysis buffer containing protease and phosphatase inhibitors using sonication. Following protein quantification by BCA assay, 50 μg total protein was separated by 4%–20% gradient SDS-PAGE and transferred to PVDF membranes using wet transfer methodology. All membranes were blocked with a TBST solution containing 5% non-fat dry milk. Subsequently, the membranes were incubated with the corresponding primary antibodies overnight at 4 °C. After washing, they were incubated with the HRP-labeled secondary antibodies (1:5000) for 1 h at room temperature. For the target proteins that needed to be detected through “membrane stripping and re-incubation”, after the first chemiluminescence imaging was completed, the membranes were treated with a mild membrane stripping buffer for 10 min to thoroughly elute the bound primary and secondary antibodies. After stripping, the membranes were thoroughly washed with TBST and the blocking step was repeated. Then, they were sequentially incubated with the primary antibodies against the next target proteins. After each stripping and re-incubation, the membranes were developed using the BeyoECL Plus chemiluminescence substrate (Beyotime, Shanghai, China; Cat #P0018S) and imaged using the SageCapture Pro 5.0 system.

The signal intensities of all protein bands were quantitatively analyzed using ImageJ software. TAAR1 expression levels were normalized to β-actin (target protein band intensity/internal reference band intensity). The expression levels of the target phosphorylated proteins were normalized to their respective total proteins and verified using β-actin as an internal reference.

The antibodies used were as follows:

TAAR1 (rabbit, 1:1000, Immunoway, Product # PA5-115999)
p-PKA (Thr197) (rabbit, 1:1000, Immunoway, Product # YP0226)
p-CREB(Ser133) (rabbit, 1:1000, GenulN Biotechnology, Product # U0177)
p-DARPP32(Thr34) (rabbit, 1:1000, Immunoway, Product # YP0950)
CREB (rabbit, 1:1000, Cell Signaling Technology, Product # 9197)
DARPP32 (rabbit, 1:1000, Immunoway, Product # YM8620)
PKA (rabbit, 1:1000, Immunoway, Product # YT3749)

### 4.6 Immunohistochemistry (IHC)

The SD rats were rendered anaesthetic with sodium pentobarbital (40 mg/kg, intraperitoneal) and subsequently perfused transcardially with Phosphate Buffered Solution (pH 7.4). Brain tissues were post-fixed in 4% paraformaldehyde for a period of 48 h, following which they underwent a series of dehydration steps using a graded ethanol solution. The tissues were then embedded in paraffin. Serial sections (4 μm) were produced and exposed to citrate buffer in a microwave oven to retrieve antigens. Endogenous peroxidase activity was quenched with 3% hydrogen peroxide, followed by blocking of non-specific binding sites using 5% normal goat serum. Sections were subsequently incubated overnight at 4 °C in a humidified chamber with primary antibody, then with species-matched horseradish peroxidase (HRP)-conjugated polymer secondary antibody for 30 min at room temperature. Following DAB chromogenic development, sections were mounted in neutral balsam. From the target brain region sections of each rat, select three representative sections with adequate spacing. Image analysis of tissue sections was conducted using ImageJ software (v2.14.0); National Institutes of Health, Bethesda, MD, United States (Varghese et al., 2014), with target protein expression levels quantitatively assessed through the H-score system (Wen et al., 2024). The final H-score value for each animal was obtained by averaging the scores from its three sections.

H-score was calculated using the formula:

$$H‐\text{score}=\sum \left[1\times \left(\%\text{weak intensity}\right)+2\times \left(\%\text{moderate intensity}\right)+3\times \left(\%\text{strong intensity}\right)\right]$$

Antibodies employed in this study:

TAAR1: Rabbit, 1:200 (Thermo Fisher Scientific, Waltham, MA, United States; Cat # PA5-115999)
p-PKA (Thr197): Rabbit, 1:200 (Immunoway, Suzhou, China; Cat # YP0226)
p-CREB (Ser133): Rabbit, 1:200 (Immunoway, Suzhou, China; Cat # YM8632)
p-DARPP-32 (Thr34): Rabbit, 1:200 (Immunoway, Suzhou, China; Cat #.YP0950)
AKT: Rabbit, 1:200 (Immunoway, Suzhou, China; Cat# YM8463)
p-AKT (Thr308): Rabbit, 1:200 (Immunoway, Suzhou, China; Cat# YP0590)
GSK3β: Rabbit, 1:4,000 (Abcam, Cambridge, United Kingdom; Cat# ab185141)
p-GSK3β (Ser9): Rabbit, 1:200 (Immunoway, Suzhou, China; Cat# YP0124)
ERK1/2: Rabbit, 1:200 (Immunoway, Suzhou, China; Cat# YM8336)
p-ERK1/2 (Thr202/Tyr204): Rabbit, 1:500 (Immunoway, Suzhou, China; Cat# YP1197)
MEK1/2: Rabbit, 1:25 (Cell Signaling Technology, Danvers, MA, United States; Cat# 4694S)
p-MEK1/2: Rabbit, 1:50 (Cell Signaling Technology, Danvers, MA, United States; Cat# 9154S)
BDNF: Rabbit, 1:500 (Abcam, Cambridge, United Kingdom; Cat# ab108319)

### 4.7 cAMP detection for TAAR1 agonist activity

The assay utilized cAMP Hunter™ CHO-K1 AGTRL1 Gi cells stably engineered to express TAAR1. The maintenance of cells was undertaken in Ham’s F-12K medium containing 10% FBS, 100 U/mL penicillin, and 100 μg/mL streptomycin. The cells were distributed into 384-well plates at a volume of 20 μL per well, following which they were subjected to a preincubation process at a temperature of 37 °C. Following the addition of test compounds at graded concentrations and a 10-min incubation, cAMP levels were quantified. Tyramine (100 μM) served as the maximal agonist control (MAX). Agonist activity was calculated as:

$$a\text{ctivity}\left(\%\right)=\frac{\left({\text{meanRLU}}_{\text{test}\;\text{sample}}‐{\text{meanRLU}\;}_{\text{vehicle}\;\text{control}}\right)}{\left({\text{meanRLU}}_{\mathrm{MAX}\;\text{control}}‐{\text{meanRLU}\;}_{\text{vehicle}\;\text{control}}\right)}\times 100\%$$

### 4.8 Dopamine2 (D_2_) receptor antagonism assay

The performance of the assays was undertaken utilising Flp-In™-CHO cells that exhibited stable expression of the D_2L_ or D_2S_ receptor. Procedures for cell culture, seeding, compound treatment, and detection were conducted as described for the TAAR1 assay. Haloperidol (10 µM) served as the maximum inhibitory control (MAX).

Inhibitory activity was calculated using the formula:

$$\text{inhibition}\left(\%\right)=\frac{\left({\text{meanRLU}}_{\text{test}\;\text{sample}}‐{\text{meanRLU}\;}_{\text{vehicle}\;\text{control}}\right)}{\left({\text{meanRLU}}_{\mathrm{MAX}\;\text{control}}‐{\text{meanRLU}\;}_{\text{vehicle}\;\text{control}}\right)}\times 100\%$$

### 4.9 5-HT_1A_ receptor agonist activity

The assays utilized 5-HT_1A_ Gα15-NFAT-bla CHO-K1 cells. These cells were cultured in MEM containing 10% FBS, 100 U/mL penicillin, 100 μg/mL streptomycin, and 600 μg/mL hygromycin B. Cells were exposed to serial dilutions of test compounds for a period of 2 hours at a temperature of 37 °C. Serotonin (5-HT, 10 µM) served as the maximal agonist control (MAX). Calcium flux was measured utilising the FLIPR® Calcium 6 Assay Kits, with agonist activity calculated as previously described for the TAAR1 assay.

### 4.10 Molecular docking

Protein-ligand interactions were analyzed using the LibDock module in Discovery Studio 2019 (Dassault Systèmes BIOVIA, San Diego, CA, United States). TAAR1 crystal structure (PDB ID: 8JLO) was preprocessed through hydrogen atom addition, charge assignment (CHARMm force field), and energy minimization (Smart Minimizer algorithm with Generalized Born implicit solvent model). The optimized receptor structure was subsequently utilized for docking. The active site was defined as the co-crystallized ligand’s binding pocket. All remaining parameters employed LibDock’s default settings.

### 4.11 Site-directed mutagenesis and functional characterization

HEK293-cAMP-biosensor-22F-H-1B2 cells in logarithmic growth phase were transfected with TAAR1 or mutant plasmids (2 μg per transfection) using jetPRIME^®^ reagent. Following 36-h incubation, cells were seeded into 96-well plates and pretreated with equilibration medium containing 2% GloSensor™ cAMP Reagent for 1 h. Subsequently, serially diluted Selutaront (initial concentration 100 μM, 3.16-fold dilutions, 10 concentrations), Forskolin (10 μM), and ZH8651 (100 μM) were administered, with triplicate wells per treatment group. Kinetic luminescence readings were acquired every minute for 30 min using a microplate luminometer to quantify compound-induced cAMP dynamics. Data were normalized to forskolin (100% cAMP response) and vehicle (0% response) controls.

### 4.12 MK-801-induced schizophrenia-like behavior

Selutaront and (+)-MK-801 hydrogen maleate were dissolved in physiological saline (0.9% NaCl) and administered at 0.1 mL per 10 g body weight.

Forty male C57BL/6J mice were allocated to four groups for the experimental phase and habituated to the behavioral testing room for 2 h prior to experimentation. Treatments were physiological saline or Selutaront (0.01, 0.03, or 0.1 mg/kg; i.g.). Baseline locomotor activity was assessed during a 30-min open field test in an arena (20 cm × 20 cm × 40 cm, length × width × height, GeneandI, Beijing, China). Following baseline assessment, all animals received MK-801 (0.8 mg/kg, i.p.), and total movement distance was quantified over a subsequent 30-min observation period.

### 4.13 Analysis of protein expression in brain tissues of MK-801 model mice

To investigate the molecular mechanism by which Selutaront antagonizes MK801-induced schizophrenia-like behaviors, the effects on the phosphorylation levels of key signaling pathway proteins were examined.

Thirty male C57BL/6J mice (6–8 weeks; 18–22 g) were randomly assigned to three groups (n = 10/group): (1) Control group: administered saline (i.g.), followed 30 min later by an intraperitoneal (i.p.) injection of saline; (2) MK-801 group: administered saline (i.g.), followed 30 min later by an i.p. injection of MK-801 (0.8 mg/kg); (3) Selutaront +MK-801 group: administered Selutaront (10 mg/kg, dissolved in saline, i.g.), followed 30 min later by an i.p. injection of MK-801 (0.8 mg/kg). Thirty minutes after the final injection (MK-801 or saline), all mice were deeply anesthetized and euthanized by decapitation. The target brain regions were rapidly dissected, flash-frozen in liquid nitrogen, and stored at −80 °C for subsequent analysis. The detailed experimental procedures for Western blotting, including tissue lysis, protein concentration determination (BCA method), electrophoresis, membrane transfer, antibody incubation, and signal detection, were consistent with the methods described in the “Western blotting” section of this article. The primary antibodies used included: rabbit anti-p-PKA (Thr197) (1:1000), rabbit anti-total PKA (1:1000), rabbit anti-p-CREB (Ser133) (1:1000), rabbit anti-total CREB (1:1000), and mouse anti-β-actin (1:5000).

### 4.14 Pharmacokinetic (PK) study

Following single-dose administration of Selutaront by oral gavage (1 mg/kg) or intravenous injection (5 mg/kg) to Sprague-Dawley rats, plasma concentrations were determined. Blood samples were collected via the retro-orbital venous plexus at the following time points post-administration: 0.083, 0.25, 0.5, 1, 2, 4, 6, 8, 12, and 24 h. The plasma concentrations of Selutaront were quantified using liquid chromatography-tandem mass spectrometry (LC-MS/MS) with an Agilent 1100 series HPLC interfaced to a TSQ Quantum Access triple quadrupole mass spectrometer (Thermo Fisher Scientific, San Jose, CA, United States). The processing of drug-concentration data was undertaken using a non-compartmental model with WinNonlin™ 6.3 (Pharsight, Mountain View, CA, United States).

Pharmacokinetic (PK) parameters, including elimination half-life (T_1/2_), maximum plasma concentration (C_max_), area under the curve (AUC), and mean residence time (MRT), were calculated.

### 4.15 Caco-2 permeability and transporter substrate assessment

Caco-2 cells were cultivated in MEM that had been enriched with 20% FBS, 100 U/mL penicillin, and 100 μg/mL streptomycin. For the purpose of transport studies, cells were seeded at 1 × 10^5^ cells/well on 96-well Transwell^®^ polycarbonate membranes and maintained for a period of 24 days to establish fully differentiated monolayers, with medium changes performed every 48 h. Test compounds were applied apically with or without verapamil (2 μM). After a 120-min incubation, samples from apical and basolateral compartments were collected. Urea content was quantified by LC-MS/MS (Agilent 1290/6470 system). Apparent permeability coefficients (Papp, cm/s) and efflux ratios (ER = Papp (B→A)/Papp (A→B)) were calculated to assess permeability and transporter involvement.

### 4.16 Evaluation of *in vitro* stability in liver microsomes

The metabolic stability of Selutaront was assessed by incubation with liver microsomes (mouse, rat, and human) and an NADPH regeneration system at 37 °C for 5, 10, 20, 30, or 60 min. The termination of reactions was achieved through the use of ice-cold acetonitrile, which was employed as the internal standard for tolbutamide. Testosterone (a CYP3A4 substrate) and dextromethorphan (a CYP2D6 substrate) were included as positive controls. The remaining concentrations of the compounds were determined using LC-MS/MS. Chromatogram processing, including retention time analysis, data acquisition, and integration for test compounds and positive controls, was performed using Analyst software (AB Sciex, Framingham, MA, United States).

### 4.17 Statistical analysis

All statistical analyses were performed using Prism 9 (GraphPad Software, La Jolla, CA, United States). Data are presented as mean ± standard error of the mean (SEM). Intergroup differences were assessed by one-way analysis of variance (ANOVA) followed by Tukey’s *post hoc* test. Statistical significance was defined as *P* < 0.05.

## 5 Conclusion

This study systematically investigated the potential of TAAR1 as a novel therapeutic target for schizophrenia and successfully developed a new, highly selective TAAR1 agonist, Selutaront. Using a TAAR1 partial knockdown rat model, we demonstrated for the first time *in vivo* that TAAR1 modulates nigrostriatal circuit function via the cAMP/PKA/CREB and DARPP-32 signaling axis, thereby influencing schizophrenia-related behaviors. *In vitro*, Selutaront exhibited potent agonist activity and excellent selectivity for human TAAR1. Furthermore, in an MK-801-induced mouse model, it dose-dependently ameliorated schizophrenia-like behaviors, an effect strictly dependent on TAAR1 expression. Further mechanistic studies confirmed that Selutaront rescues the function of the cAMP/PKA/CREB signaling pathway by reversing MK-801 induced suppression of PKA and CREB phosphorylation, thereby mediating its antipsychotic-like effects. Additionally, Selutaront demonstrated favorable oral bioavailability, low efflux risk, high metabolic stability, and excellent brain penetration, providing a solid pharmacokinetic foundation for its clinical translation.

## Data Availability

The original contributions presented in the study are included in the article/Supplementary Material, further inquiries can be directed to the corresponding authors.
