# Supplementary material for: TAAR1-mediated pathways regulating nigrostriatal function and the discovery and pharmacological characterization of a novel TAAR1 agonist, Selutaront
Source: Front Pharmacol. 2026 Feb 23;17:1759454. doi: 10.3389/fphar.2026.1759454 (PMC12967949; doi:10.3389/fphar.2026.1759454)
Supplement: Supplementary file 1 [file DataSheet1.docx]

Supplementary Material


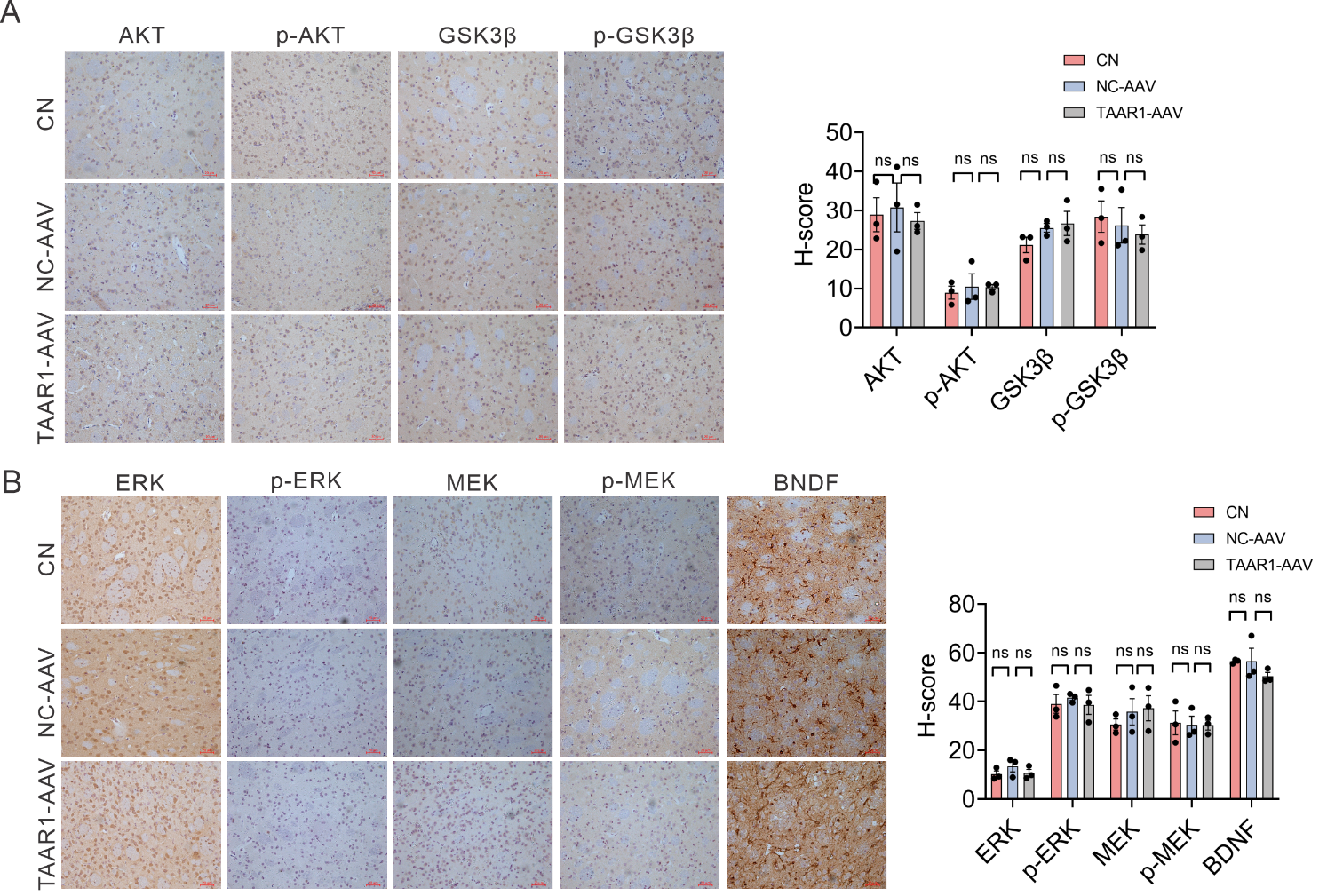


**Supplementary Figure 1.** Effect of TAAR1 knockdown on the expression of AKT/GSK-3β and other related signaling pathway proteins in rat striatum. Representative images and quantitative analysis of p-AKT, total AKT, p-GSK-3β, and total GSK-3β (A) and p-ERK1/2, total ERK1/2, p-MEK1/2, total MEK1/2, and BDNF (B) in the striatum of rats.


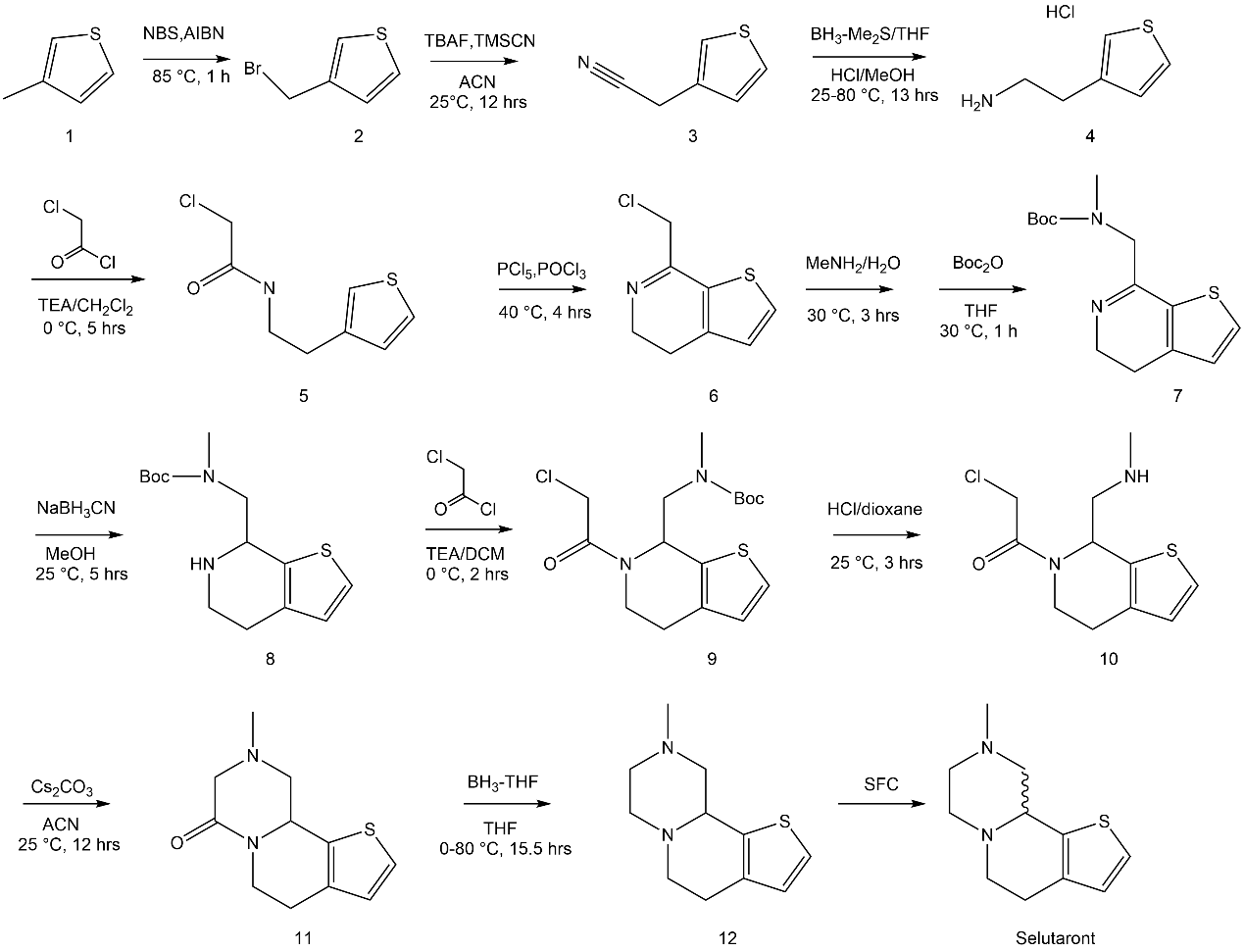


**Supplementary Figure 2.** Synthesis of Selutaront.

**Supplementary Figure 3.** 1HNMR (400 MHz, DMSO-d_6_) spectrum of Selutaront: 7.92 - 7.78 (m, 1H), 7.57 - 7.51 (m, 2H), 7.45 - 7.24 (m, 9H), 6.46 - 6.37 (m, 1H), 5.41 - 5.35 (m, 4H), 1.33 - 1.20 (m, 12H)


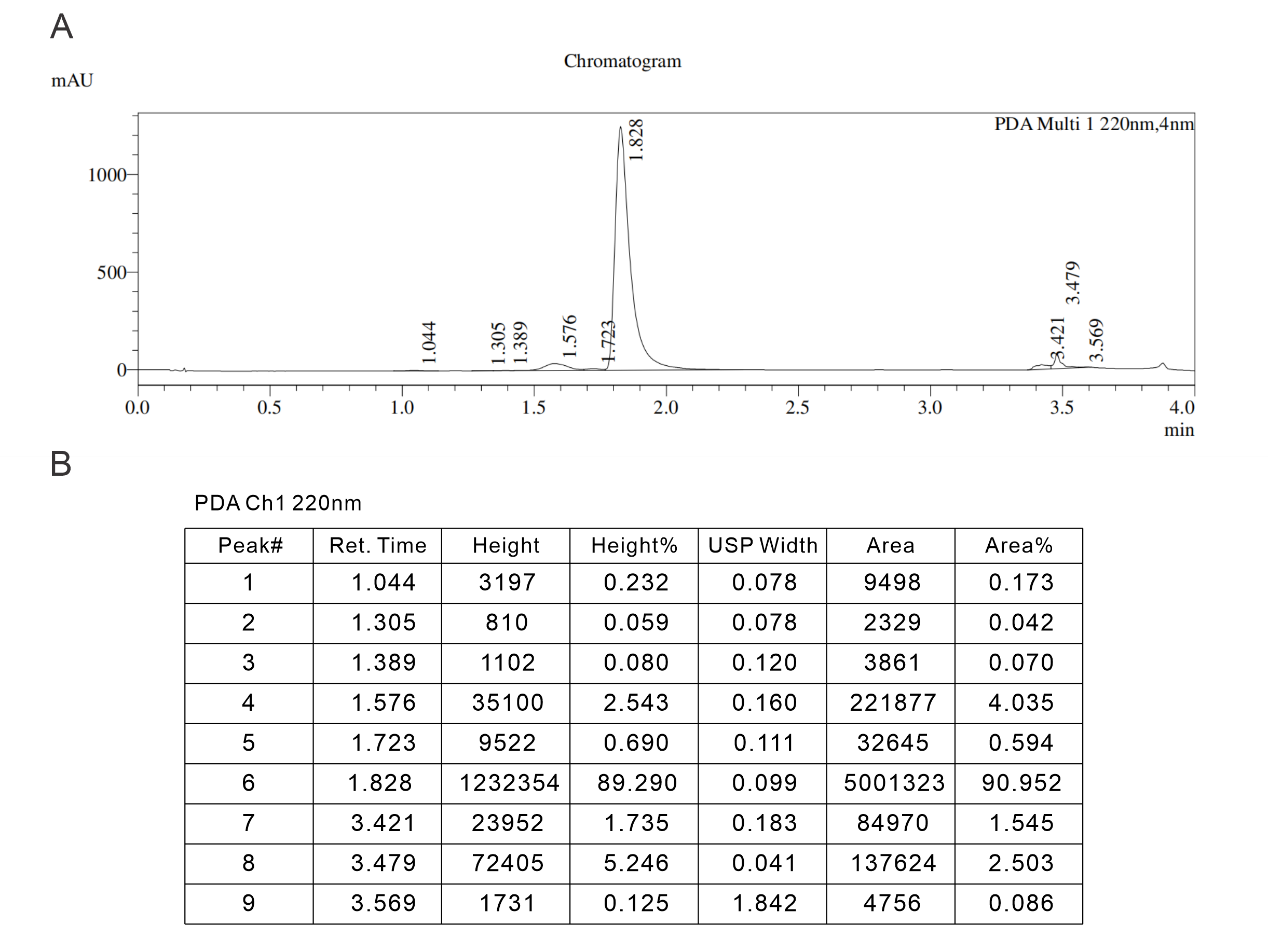


**Supplementary Figure 4.** LC-MS analysis of Selutaront: MS (ESI) m/z = 209.1 [M+H] ^+^


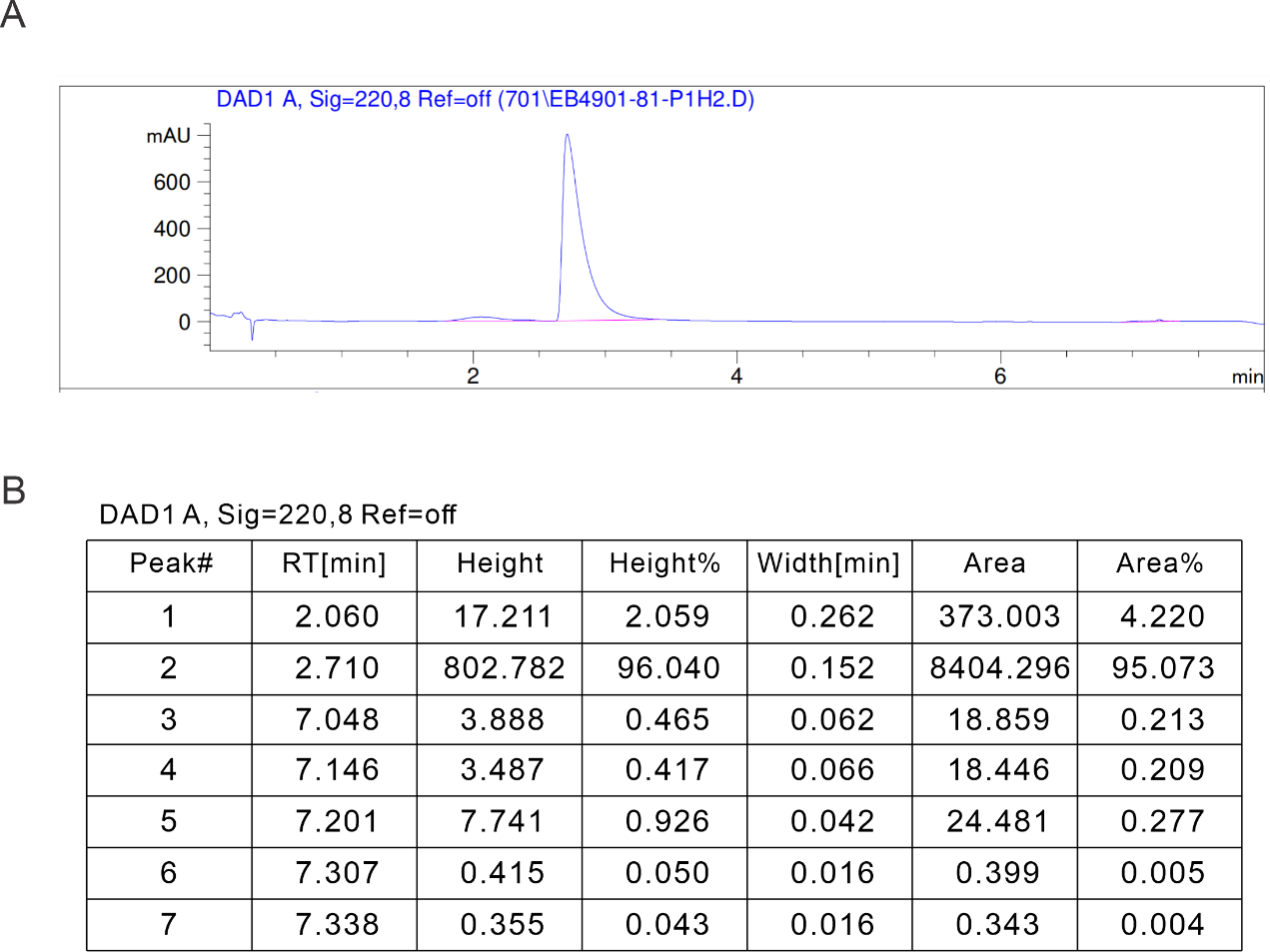


**Supplementary Figure 5.** HPLC analysis of Selutaront
